# Killing of Coccidioides by drugs

**DOI:** 10.1099/mic.0.001666

**Published:** 2026-02-12

**Authors:** David A. Stevens

**Affiliations:** 1 Division of Infectious Diseases and Geographic Medicine, Stanford Univ., Medical School, Stanford, CA 95304, USA

**Keywords:** *Coccidioides*, drug susceptibility, minimum fungicidal concentration

## Abstract

Coccidioidomycosis has long been regarded as one of the most difficult mycoses to eradicate in patients with progressive infection. There are few published studies of inhibition of *Coccidioides in vitro*, comparing the results with various drugs as measured by minimal inhibitory concentration (MIC). An ability to kill *Coccidioides*, at achievable concentrations in the host, may be necessary to produce a cure. Five hundred fifty-seven killing assays of *Coccidioides* clinical isolates *in vitro*, with minimum fungicidal concentrations (MFCs), are reported here. Heterogeneity of MFCs for all drugs is shown. Thus, many antifungals can kill *Coccidioides*, in addition to just inhibiting growth. The drug most commonly requiring the highest concentrations for killing was fluconazole, and posaconazole generally the lowest concentrations tested. For all the major drugs in use, some MFCs could not be exceeded by achievable blood (and presumably tissue) concentrations by usual dosing methods. MFCs were overwhelmingly within one twofold dilution of the MIC, but for ~5% of the major drugs, MFCs were ≥8-fold higher than the MIC. The distribution of the MFCs shown for each drug, and particularly the probability of having one of the minority of isolates with a high MFC value, might be of interest to the clinician in choosing a drug or its dose, particularly if susceptibility results will not be available, or before susceptibility results are available. MIC or MFC results *in vitro* are but a starting place for correlations with *in vivo* outcome in therapy. MFCs could be explored for incorporation into pharmacodynamic indices.

## Introduction

Coccidioidomycosis is of particular interest presently because of the large increase in incidence [1] and its geographic spread [2]. Although inhaling *Coccidioides* most commonly results in an infection that is mild, often unrecognized [3] or even asymptomatic [4], when the infection persists as a chronic or progressive pulmonary condition, or disseminates, the mycosis has been regarded as among the most difficult to treat, even in non-immunocompromised hosts. In immunocompromised hosts, such infection is frequently fatal, even with antifungal treatment [5]. The refractoriness could be related to the conversion *in vivo* from arthroconidia (environmental phase) to spherules (parasitic phase) [6]. Spherules are large forms, difficult for phagocytes to ingest, and spherules have antiphagocytic external defences [7]. Coccidioidal parenchymal lesions are characteristically suppurative and show granuloma formation, both of which retard antifungal drug penetration [8].

Evidence for the difficulty of treatment includes the extraordinary length of therapy recommended by experts for the progressive forms of the disease [9]. Related to that, in the randomized trial of coccidioidal therapy (in noncompromised hosts) [10], a clear-cut difference was only evident when observation was extended to 12 months of therapy, as opposed to the initial intent to analyse at 9 months. Furthermore, even after apparently successful therapy on clinical grounds, the fungus appears to persist lifelong in viable form, as is evident from reactivations in asymptomatic hosts who have left the endemic areas (thus excluding re-infection) but then become immunocompromised because of another disease or its treatment and develop reactivated infection [5]. Further evidence for long persistence of viable *Coccidioides* is in resected tissue specimens from asymptomatic patients (e.g. lung nodule discovered on radiograph) [11].

Amphotericin B was the first effective anticoccidioidal therapy introduced, but as new azole drugs were successively investigated in anticoccidioidal therapy (including those for which this author was a participant), it is noteworthy that the patient cohorts available for these studies were commonly patients who had failed prior therapy or relapsed after apparently successful therapy (in addition to those in whom side effects of prior therapy had truncated courses of therapy) [12,18]. In one form of disseminated disease, meningitis, it is clear that lifelong azole therapy is required [19,20].

There are a few published studies of inhibition of *Coccidioides in vitro*, comparing the results with various drugs as measured by MIC (minimal inhibitory concentration) [21,25; G.R. Thompson *et al*., ms. in preparation]. Aside from our study, including only three coccidioidal isolates [26], I am aware of only one other study that purportedly examined the ability of antifungals to kill *Coccidioides in vitro*, as measured by established laboratory methods to examine minimal fungicidal concentrations (MFCs), and that study [23] presented no MFC data in the text, only briefly summarizing it in the Abstract section. An ability to kill *Coccidioides*, at achievable concentrations in the host, may be necessary to produce a cure, especially when the host immune response is impaired. This study extensively examined MFC determinations on clinical *Coccidioides* isolates.

## Methods

### Isolates and testing plan

These were *Coccidioides* isolates, in the mycelial form, sent to my research laboratory at the California Institute for Medical Research from 1991 to 2023, which also maintained a clinical laboratory licence and had Biohazard level 3 certification (required to handle coccidioidal isolates). These were sent by clinicians, >90% from California, desiring to know susceptibility results. However, it is almost always impossible, except in instances of focal epidemiological outbreaks, to know in what location the patient acquired their coccidioidal infection. Limited clinical information about the patients was available, but overwhelmingly, the requests appeared to have originated prior to the start of any prolonged therapy course. The patients' subsequent course was, in most instances, unknown to us. The exception to clinician test initiation was that some clinical isolates were tested at the request of pharmaceutical companies, who wished to test their products during their preclinical or early clinical phase and, in some instances, to know how their products compared with drugs already marketed and which had not already been tested against the latter group of drugs.

The isolates studied were 1 per patient, and 1 to 6 MFC drug assays were performed on each isolate (total, 557 MFC assays on 140 isolates, average 4 tests/isolate). The exception to ‘one per patient’ was three isolates from the same patient, the second and third obtained later in his course after various treatments (the re-isolates had the same susceptibility results as the initial one). MICs were determined on all isolates tested, and MFCs if requested. The drugs tested were at the request of the sending physician, hospital, clinic and, in a few instances, a pharmaceutical company. The MIC data on all these isolates are part of an extensive manuscript in preparation, comparing our laboratory results with those of the Univ. of Texas, San Antonio laboratory (where only MICs were tested), and also examining temporal MIC trends in both laboratories (G.R. Thompson *et al.*, manuscript in preparation).

### Testing methodology

Broth macrodilution was performed in tubes for MIC and MFC determinations as previously described [26,29]. In brief, arthroconidia were harvested from mycelia and suspended in broth. Inoculum (0.1 ml/tube) was dispensed to each tube. Pure drug powders were obtained from the manufacturers. The range of drug concentrations to be tested was set to reflect known pharmacokinetics in humans of the drugs to be tested; these ranges are shown and discussed in the Results section. At times, pharmacokinetics were unknown at the initiation of testing with a drug, and then, a very broad range of drug concentrations was instituted. The drug was diluted in twofold steps and combined with the inocula in each tube for a final volume of 2 ml and a final concentration of 10^3^ arthroconidia per millilitre. Before incubation, all tubes are clear. A novel feature, as viewed presently, was the medium used throughout for the studies reported, SAAMF [30], chosen because it is fully defined (and lacking any components that could antagonize drugs, particularly new ones with as yet undefined mechanisms of action) and because we have found that all *Coccidioides* isolates grow well in it. A drug-free tube (inoculum growth control), an inoculum-free tube (reagent sterility control) and tubes containing the drug to be tested but with a known pansensitive *Candida kefyr* isolate (drug potency control) were concurrently established. Reproducibility of results was established annually with a sample of isolates, per California State Dept. of Health and Federal standards and inspections. Testing of amphotericin B is, in all testing laboratories, performed with the deoxycholate form, as amphotericin B is the active principle in the lipid-formulated amphotericin B preparations as well [28].

The tubes were incubated at a temperature to approximate human body temperature (35–37 °C) for 1 week (at this time, the control in the drug-free tube was deemed 4+/4, semiquantitatively), and the MIC was determined by the clear tube at the lowest drug concentration. Tubes with less drug than that will show turbidity, thus lack of inhibition and no killing. After vortex-mixing, 50 microlitres were removed from the clear tubes and cultured on SAAMF agar for 1 week at that temperature, and the agar was enumerated for coccidioidal colonies. If no colonies, this implied, based on this sampling, ≥99% killing (MFC). It is obvious that MFC could not, by definition, be lower than MIC, since clear tubes were the substrate for the MFC determinations.

## Results

### Amphotericin B

The initial systemic antifungal drug applied to coccidioidomycosis was amphotericin B, as mentioned previously. The range for testing was set at 0.125–8 mcg ml^−1^, reflecting the fairly low Cmax values after parenteral administration of amphotericin B deoxycholate [31,32]. The results (Table 1) show a fairly even spread over the range of testing, indicating considerable heterogeneity in the fungal population.

**Table 1.** MFCs for amphotericin B

**mcg ml^−1^**	0.25	0.5	1	2	4	≥8
Number of isolates with result (**% of total tested**)	1 (**1**%)	6 (**7**%)	23 (**25**%)	23 (**25**%)	21 (**23**%)	19 (**20**%)

Amphotericin B (*n*=93 isolates).

#### Fluconazole

One of the first three oral azoles to be studied in coccidioidomycosis was fluconazole. The range for testing was set at 0.78–100 mcg ml^−1^, reflecting the relatively high blood levels potentially achievable with this drug, particularly with aggressive dosing that is possible with relatively few adverse effects [15,31, 32]. The results (Table 2) show a clustering towards the high end of the range. Heterogeneity is evident. This profile needs to be compared to the other azoles, whose results now follow.

**Table 2.** MFCs for fluconazole

**mcg ml^−1^**	≤0.78	1.56	3.13	6.25	12.5	25	50	≥100
Number of isolates with result (**% of total tested**)	1 (**1**%)	0	1 (**1**%)	6 (**5**%)	23 (**18**%)	26 (**20**%)	28 (**22**%)	44 (**36**%)

Fluconazole (mcg ml−1) (*n*=128 isolates).

### The other major antifungal azoles

Itraconazole was introduced into anti-coccidioidal therapy about the same time as fluconazole. Isavuconazole was the last of this group to be so employed. The range tested (0.39–12.5 mcg ml^−1^) reflects the narrower range of blood levels achieved with this group of four drugs (Table 3).

**Table 3.** MFCs for four major azole antifungals

**mcg ml^−1^**	≤0.39	0.78	1.56	3.13	6.25	≥12.5
**Itraconazole**	***n*=102**					
	21 (**21**%)	29 (**28**%)	18 (**27**%)	12 (**12**%)	7 (**7**%)	5 (**5**%)
**Posaconazole**	**(*n*=74)**					
	32 (**47**%)	14 (**19**%)	14 (**19**%)	8 (**11**%)	1 (**1**%)	2 (**3**%)
**Isavuconazole**	**(*n*=30)**					
	2 (**7**%)	7 (**23**%)	13 (**43**%)	8 (**27**%)	0	0
**Voriconazole**	**(*n*=60)**					
	17 (**28**%)	14 (**23**%)	19 (**32**%)	4 (**7**%)	1 (**2**%)	5 (**8**%)

Number of isolates with result, for each drug (% of total tested).

Heterogeneity within each drug is evident. Of the antifungals presented thus far, posaconazole is remarkable in that nearly half of the isolates tested were susceptible ‘off the lowest end’ of this scale. As it was curious to know how low these posaconazole ≤0.39 mcg ml^−1^ MIC and MFCs might actually be, for one such isolate, several more twofold dilutions of drug were prepared to determine an actual endpoint. The MIC for this isolate was 0.02 mcg ml^−1^, 16-fold lower than the ≤0.39 mcg ml^−1^ result might have suggested.

The distribution of MFCs for itraconazole and voriconazole is remarkably similar. Killing by isavuconazole appears only slightly less potent in general, on a mcg ml^−1^ basis, than these two other azoles. The contrast of all four of these drugs with the distribution compared to fluconazole (Table 2) is striking.

### Other antifungals and other drugs

Ketoconazole was often used when first introduced in coccidioidal therapy [13], prior to itraconazole and fluconazole, but fell into disuse when the other azoles were introduced, largely because of perceived adverse effect issues with ketoconazole. A few isolates could be tested before the other azoles displaced it (Table 4). The number of isolates there is small, but the distribution appears similar to posaconazole, suggesting similar potency.

**Table 4.** MFCs for another azole antifungal and for echinocandins

**mcg ml^−1^**	≤0.39	0.78	1.56	3.13	6.25	≥12.5
**Ketoconazole**						
***n*=7**	3 (**43**%)	1 (**14**%)	1 (**14**%)	2 (**29**%)	0	0
**Echinocandins**						
**Caspofungin**						
***n*=15**	0	1 (**7**%)	1 (**7**%)	2 (**13**%)	2 (**13**%)	9 (**60**%)
**Anidulafungin**						
***n*=7**	0	1 (**14**%)	2 (**29**%)	1 (**14**%)	2 (**29**%)	1 (**14**%)
**Micafungin**						
***n*=4**	0	0	0	0	0	4 (1**00**%)

Number of isolates with result, for each drug (% of total tested).

The echinocandins are rarely used in coccidioidal therapy, but a few isolates have been tested (Table 4), with a suggestion of weak killing power, although for a few isolates, it is suggested that glucan synthase [33] (or an alternative echinocandin target) may be a viable target for some coccidioidal isolates.

In addition (not shown in tables, starting twofold dilutions for this next group were 100 or 6.25 mcg ml^−1^), 12 coccidioidal isolates had MFCs performed for flucytosine, and all the results were >100 mcg ml^−1^, indicating no activity. Three isolates were tested against UR-9746 and UR-9751, with MFCs>100 for both azoles. Two isolates were tested against sertraline, with an MFC of 6.25 mcg ml^−1^ for one and ≥6.25 mcg ml^−1^ for the other. One isolate was tested against methotrexate and one against griseofulvin, with MFCs of ≥6.25 and >50 mcg ml^−1^, respectively.

With starting dilutions of 8 mcg ml^−1^, two coccidioidal isolates were tested against nystatin, with an MFC of ≥8 mcg ml^−1^ for both. Five isolates were tested against a preclinical azole, ITF2534, with an MFC of 8 mcg ml^−1^ for all, and one against a preclinical non-azole, GL 048656, with an MFC of 2 mcg ml^−1^. With a starting dilution of 10 mcg ml^−1^, 7 isolates were tested against the preclinical non-azole nikkomycin Z [34], with an MFC of 2.5 mcg ml^−1^ for 1 and ≥10 mcg ml^−1^ for 6 isolates.

### How closely does the MFC follow the MIC?

All MFC determinations required an MIC determination, as the first clear tube at time of reading (the MIC), and tubes with greater drug concentrations, was subcultured to determine the MFC. Isolates tested for both MIC and MFC are a subset of the total MIC request results (the latter not a subject of this study; G.R. Thompson *et al.*, manuscript in preparation) for which the MFC was also requested. For example, the MFC results in Tables 1–3, for the 6 major anticoccidioidal drugs, are 487 MFC assays for the 1,050 total MIC assays requested (46%) for those drugs. It was of interest to examine the discrepancy between the MIC and MFC determined, not only the MFC profiles for the various drugs (Tables 1–4): how much more drug was required to kill the isolate, how good a surrogate the MIC is for the MFC and how much more information was gained from the MFC determination. This information is given in Table 5. When an MIC and/or an MFC could not be precisely determined because the result was beyond the upper end of the range tested, in those few instances, the difference between the MIC and MFC could not be precisely determined or could only be shown as an ‘≥’ value. Thus, the total number of assays in Table 5 is slightly less than the totals in Tables 1–4.

**Table 5.** Differences of the MFC exceeding the MIC

Drug	Isolates	MFC same as MIC	**2-fold**	≥2-fold	**4-fold**	**8-fold**	≥8-fold	**16-fold**	≥16-fold	≥32-fold
Amphotericin B	87	74 (**85**%)	4 (5%)	1 (1%)	3 (3%)	1 (1%)	2 (**2**%)	1 (1%)	1 (**1**%)	0
Fluconazole	99	69 (**70**%)	19 (**19**%)	5 (5%)	1 (1%)	2 (**2**%)	1 (1%)	1 (1%)	0	1 (1%)
Itraconazole	99	66 (**67**%)	23 (**23**%)	0	5 (5%)	2 (2%)	0	1 (1%)	2 (2%)	0
Posaconazole	71	60 (**81**%)	6 (**9**%)	0	3 (4%)	2 (3%)	0	0	0	0
Isavuconazole	30	20 (**67**%)	10 (**33**%)	0	0	0	0	0	0	0
Voriconazole	55	40 (**73**%)	15 (**27**%)	0	0	0	0	0	0	0
Ketoconazole	7	6 (**86**%)	1 (**14**%)	0	0	0	0	0	0	0
Caspofungin	12	10 (**83**%)	2 (**16**%)	0	0	0	0	0	0	0
Anidulafungin	7	7 (100%)	0	0	0	0	0	0	0	0
ITF2534	5	5 (100%)	0	0	0	0	0	0	0	0
Sertraline	1	1 (100%)	0	0	0	0	0	0	0	0
GL048656	1	1 (100%)	0	0	0	0	0	0	0	0
Nikkomycin Z	1	1 (100%)	0	0	0	0	0	0	0	0

Number of isolates compared (% in bold). Some isolates shown as ‘(≥)’ were not titrated to the ultimate endpoint.

As Table 5 shows, for every drug, the MIC=MFC for 67–100% of the isolates. Thus, this says that at concentrations of a drug that could inhibit sufficiently to produce a clear tube (MIC), that concentration was highly likely to also kill the isolate. It is achieving that concentration *in vivo*, at the site of infection, that is the issue. If the comparison lumped the instances where MIC=MFC, plus when the MFC was only twofold greater than the MIC (columns 3 and 4, Table 5), the congruence was 89–100% of all isolates and MFC tests for all drugs. These congruences were even true for fluconazole and amphotericin B, whose MFCs tended to be at the upper end of the ranges tested (Tables 1–2). However, the value of knowing the MFC may be demonstrated for the minority of isolates where the MIC–MFC discrepancy was large. For example, where the MFC was ≥8-fold higher than the MIC, there were ~5% of the isolates tested, for each of 3 of the 6 major drugs (Table 5). Not shown in Table 5 are the results with UR-9746 and UR-9751 [26], where the six MFCs were three- to sevenfold higher than the corresponding MIC.

## Discussion

The tables show that many antifungals can kill *Coccidioides*, in addition to just inhibiting growth (MIC; G.R. Thompson *et al.*, manuscript in preparation). The distribution of the MFCs for each drug in the tables, and particularly the probability of having one of the minority of isolates with a high MFC value, shown here might be of interest to the clinician in choosing a drug or its dose, particularly if susceptibility results will not be available, or before susceptibility results are available*.*

The distribution of results for the principal drugs (Tables 1–3) is grouped near the lower end of the range tested for all, although that is less the case for amphotericin B, and particularly fluconazole. A broad range of susceptibilities for many drugs has previously been noted for coccidioidal MICs [25]. With respect to patterns for individual isolates, none could be seen, among the total studies here. That is, analysis of MFC studies on individual isolates, when >1 MFC study on the same isolate was requested, revealed no instance where the high-end values were seen for all the different MFCs ordered.

Amphotericin B has already fallen into disfavour because of its side effects [35], and the comparative ease of using oral drug alternatives, and there is growing disenchantment in several centres with fluconazole for coccidioidomycosis, particularly for certain clinical presentations of disseminated disease [20,36], reflected in recommendations for using higher doses [20] than initially usually employed, as a result of observations of clinical inefficacy. Starting doses of up to 1,200 mg of fluconazole have been recommended [9]. The inferiority of fluconazole to other azoles against *Coccidioides*, with respect to MICs, has previously been described in comparisons with itraconazole [23–25; G.R. Thompson *et al.*, manuscript in preparation], posaconazole [37], isavuconazole [38] and with some other azoles [23,25]. One report also preliminarily suggested that this is true for fluconazole vs. other azoles for MFCs [23]. A study of MICs [25] has also noted a small percent of isolates with elevated values to major azoles in clinical use, as we have noted presently with MFCs. A dominance of high MICs in caspofungin testing has been found previously [22,25]. Generally, low ketoconazole MICs have been noted [23].

Posaconazole appears to be generally the most potent in the present MFC study, on a mcg ml^−1^ basis. Excellent results with posaconazole therapy have been reported in coccidioidal animal models [37], in patients [16] and even in clinical salvage therapy of failures from prior other therapies [17].

### Can the Cmax achievable with these drugs exceed these MFCs?

Recent literature reviews indicate that the Cmax (peak serum concentration) range for adults for these drugs, at usual doses, is shown in Table 6 [31,32]. There is, of course, some variation (as well as dependency on dose administered, as well as Cmax after single or multiple doses), than what is shown in the table, in these Cmax values among different investigators, and these concentrations will be lower in most instances owing to problems of absorption of oral agents in some patients, higher with some drugs in cases of renal dysfunction and higher or lower owing to drug–drug interactions with other agents given to patients. Moreover, intraparenchymal concentrations at the site of infection would be expected to be lower than the Cmax [31,32], calculated from blood, which is another gradient testing the ability of drugs with the given MFCs to effect a cure. Note that the later introduction of lipid-formulated amphotericin markedly alters the Cmax achievable for that drug.

**Table 6.** Cmax from literature reviews

Drug	Cmax (mcg ml^−1^)
Amphotericin B deoxycholate	0.5–5.4
Liposomal amphotericin B	14–47
Amphotericin B lipid complex	1.6
Fluconazole*	4.1–20
Itraconazole	0.3–1.3
Posaconazole	0.6–4
Isavuconazole	0.5–2.6
Voriconazole	2–4.6
Caspofungin	7–10
Anidulafungin	7
Micafungin	3.4–18

*With 400 mg oral dose.

Pharmacodynamic indices that need further exploration in predicting outcome in coccidioidomycosis include, among others, Cmax or AUC divided by the MIC, and it is suggested here that, alternatively, exploring MFC as the denominator might give valuable information, particularly with some isolates with unusually high MFCs, or when MIC and MFC are not closely linked (Table 5). It appears evident that given the MFCs determined, for some drugs (e.g. amphotericin B deoxycholate and fluconazole), it is unlikely, even with radical dosing regimens, that many MFCs would be exceeded by drug concentrations in any body compartment, and this is also true for a minority of the isolates for the other drugs, where some MFCs appear towards the high end of the dilution series. Such a disparity between fluconazole susceptibility and blood levels achievable with ordinary dosing regimens has also been suggested with regard to many MICs in prior studies [23,25]. Although the duration of exposure in infected organs may rescue a favourable outcome even when MFCs appear to be insuperable, the prevalence of MFCs exceeding Cmax may explain the difficulties in achieving true cure *in vivo*, as has been discussed in the Introduction section. Moreover, with a drug such as amphotericin B deoxycholate, most likely of all to produce truncated courses of therapy because of side effects [35], it might be most difficult to sustain desirable concentrations in tissue. The lipid-formulated amphotericin preparations are not without side effects, and the requirement for parenteral therapy for those is also limiting.

### Shortcomings of the present study

There are two species of *Coccidioides*, *Coccidioides immitis* and *Coccidioides posadasii*, and as our isolates are overwhelmingly from California, *C. immitis* likely is predominant [39]. However, no differences in drug susceptibilities between the two species have been found [23]. It is important to note that MIC or MFC results are but a starting place for correlations, in animal models [40] or in patients, to derive predictions about efficacy, and the effect of various dosing regimens. Imputing importance to MFC results (or for MICs, for that matter) does not account for host immune contribution to killing (or inhibition) [41]. However, drug activity alone is likely more critical if the patient is immunocompromised [5]. Moreover, the present study, as for similar prior studies on MICs, has not considered the possibility of achieving either greater inhibitory or greater killing power as a result of synergy between antifungals [42]. The present study was performed on mycelial *Coccidioides*, not the parasitic phase. This is because only arthroconidia of the mycelial phase can logistically be tested (because of difficulties in growing spherules and endospores *in vitro*, and even greater difficulties in quantitating an inoculum of those elements). This limitation is true of all the prior coccidioidal drug testing *in vitro* in the literature. It is unclear whether there are differences in phase susceptibilities for an individual isolate. Lastly, it would be important to identify the genomic determinants of reduced susceptibility and to determine whether resistance can develop during therapy.

## Abbreviations

MFCs: minimum fungicidal concentrations
MIC: minimal inhibitory concentration
